# rGO/Silk Fibroin-Modified Nanofibrous Patches Prevent Ventricular Remodeling *via* Yap/Taz-TGFβ1/Smads Signaling After Myocardial Infarction in Rats

**DOI:** 10.3389/fcvm.2021.718055

**Published:** 2021-08-16

**Authors:** Yanjing Feng, Guoxu Zhao, Min Xu, Xin Xing, Lijun Yang, Yao Ma, Mengyao Qi, Xiaohui Zhang, Dengfeng Gao

**Affiliations:** ^1^Department of Cardiology, The Second Affiliated Hospital, Xi'an Jiaotong University, Xi'an, China; ^2^School of Material Science and Chemical Engineering, Xi'an Technological University, Xi'an, China; ^3^The Key Laboratory of Biomedical Information Engineering of Ministry of Education, School of Life Science and Technology, Xi'an Jiaotong University, Xi'an, China

**Keywords:** reduced graphene oxide, acute myocardial infarction, myocardial fibrosis, cardiofibroblasts, YAP/TAZ-TGFβ1/Smads signaling

## Abstract

**Objective:** After acute myocardial infarction (AMI), the loss of cardiomyocytes and dysregulation of extracellular matrix homeostasis results in impaired cardiac function and eventually heart failure. Cardiac patches have emerged as a potential therapeutic strategy for AMI. In this study, we fabricated and produced reduced graphene oxide (rGO)/silk fibroin-modified nanofibrous biomaterials as a cardiac patch to repair rat heart tissue after AMI and investigated the potential role of rGO/silk patch on reducing myocardial fibrosis and improving cardiac function in the infarcted rats.

**Method:** rGO/silk nanofibrous biomaterial was prepared by electrospinning and vacuum filtration. A rat model of AMI was used to investigate the ability of patches with rGO/silk to repair the injured heart *in vivo*. Echocardiography and stress–strain analysis of the left ventricular papillary muscles was used to assess the cardiac function and mechanical property of injured hearts treated with this cardiac patch. Masson's trichrome staining and immunohistochemical staining for Col1A1 was used to observe the degree of myocardial fibrosis at 28 days after patch implantation. The potential direct mechanism of the new patch to reduce myocardial fibrosis was explored *in vitro* and *in vivo*.

**Results:** Both echocardiography and histopathological staining demonstrated improved cardiac systolic function and ventricular remodeling after implantation of the rGO/silk patch. Additionally, cardiac fibrosis and myocardial stiffness of the infarcted area were improved with rGO/silk. On RNA-sequencing, the gene expression of matrix-regulated genes was altered in cardiofibroblasts treated with rGO. Western blot analysis revealed decreased expression of the Yap/Taz-TGFβ1/Smads signaling pathway in heart tissue of the rGO/silk patch group as compared with controls. Furthermore, the rGO directly effect on Col I and Col III expression and Yap/Taz-TGFβ1/Smads signaling was confirmed in isolated cardiofibroblasts *in vitro*.

**Conclusion:** This study suggested that rGO/silk improved cardiac function and reduced cardiac fibrosis in heart tissue after AMI. The mechanism of the anti-fibrosis effect may involve a direct regulation of rGO on Yap/Taz-TGFβ1/Smads signaling in cardiofibroblasts.

## Introduction

Acute myocardial infarction (AMI) is a common cardiac emergency, with potential for substantial morbidity and mortality; more than 7 million people have infarctions each year (1, 2). After AMI, the loss of cardiomyocytes and dysregulation of extracellular matrix (ECM) homeostasis results in impaired cardiac function and leads to heart failure (3, 4). With the development of therapies, the early mortality rate with AMI has declined, but the incidence and prevalence of post-MI heart failure continues to increase (5). Recently, accompanied by the rapid development of cardiac tissue engineering, different kinds of cardio-supportive devices have been manufactured as new strategies for cardiac tissue repair after AMI (6–8). On the basis of the structural and biological properties of myocardial tissue (9, 10), a composite biomaterial with good mechanical support, biocompatibility and excellent biological function is needed for preparing cardiac patches to repair cardiac tissue after AMI.

Silk fibroin (SF) is a natural biopolymer derived from *Bombyx mori* cocoons, it has unique biocompatibility, biodegradability, morphologic flexibility and a number of tangible mechanical properties (11). Many studies have shown promising results for SF-based scaffolds to regenerate cardiovascular tissue both *in vitro* and *in vivo* (12–15). For instance, Chen et al. demonstrated that a Chitosan/SF-modified nanofibrous patch grafted on the infarcted myocardium could be integrated with native cardiac cells and promote cardiac function (14). Recently, reduced graphene oxide (rGO) has been regarded as one of the widely investigated nanomaterials with excellent physical, chemical properties and multiple biological functions (16–20). Furthermore, rGO-based biomaterials including bone, nerves, muscle, and cardiac tissue have been wildly used in tissue engineering technology (21, 22). We previously developed rGO-functionalized nanofibrous biomaterials that showed a great ability to promote cardiomyocyte structure formation and functions *in vitro*, including the expression of cardiac-specific proteins, formation of sarcomeric structures and gap junctions, and spontaneous beating of regenerated cardiac tissues (23). However, further study was needed to explore the role of rGO/silk nanofibrous biomaterials *in vivo*.

In the present study, rGO/silk nanofibrous biomaterials were incorporated in aseptic cardiac patches and used to repair rat heart tissue after AMI. Masson's trichrome staining, immunohistochemistry and myocardial stiffness were used to observe myocardial fibrosis at 28 days after cardiac patch implantation. From these results, we then explored and validated the potential molecular mechanism of rGO reducing cardiac fibrosis in the infarcted zone. Illuminating the role of rGO in ameliorating AMI-induced cardiac remodeling may help in understanding the underlying mechanism and suggest a new therapeutic method.

## Materials and Methods

### Fabrication of the rGO/Silk Nanofibrous Patches

rGO/silk nanofibrous scaffolds were obtained by coating and reducing the GO membrane with silk nanofibrous mats, which was synthesized as described (23). Briefly, the 8% (wt/v) silk solution and 5% (wt/v) poly (ethylene oxide) (PEO, 900 000 MW, Sigma-Aldrich, St. Louis, MO) was mixed in a ratio of 4:1 and stirred for 15 min at room temperature. Next, the prepared silk solution underwent electrospinning at a voltage of 10 kV to obtain silk nanofibers, and the electrospun nanofibers were collected on a high-speed rotating disc collector. Then the GO solution at 0.02 mg/ml was conglutinated onto the surface of silk nanofibrous mats. Finally, the GO/silk material was immersed in a 1% (wt/v) ascorbic acid (Sigma-Aldrich, St. Louis, MO) solution at 95 °C for 60 min to obtain rGO/silk materials.

### Construction of AMI Model and Implantation of rGO/Silk Patches

The experimental protocol used in the present study was approved by the Institutional Animal Care Committee at Xi'an Jiaotong University. A total of 40 male Sprague-Dawley rats (8-week-old male, Laboratory Animal Center of Xi'an Jiaotong University, China) weighing 200–220 g were used. Rats were randomly divided into four groups: sham (*n* = 10), MI (*n* = 10), MI+ silk patch (*n* = 10) and MI+ rGO/silk patch (*n* = 10). Rats were anesthetized with 2% (w/v) sodium pentobarbital (30 mg/kg) by intraperitoneal injection. Then rats underwent endotracheal intubation and assisted ventilation (tidal volume, 3 ml/100 g body weight; ventilation rate, 80/min) and the heart was exposed through a left-sided open thoracotomy. The left anterior descending coronary artery was ligated with 6-0 polypropylene suture. Myocardial ischemia was confirmed by regional cyanosis and ST-segment elevation on electrocardiography. The rats in the sham group underwent only exposure of the heart, without artery ligation. Silk patches and rGO/silk patches (8^*^8 mm^2^) were sutured onto the left ventricular epicardial surface. Then, the thoracotomy was closed in multiple layers.

### Echocardiography for Cardiac Function

Echocardiography was used to evaluate the cardiac function of rats at 28 days after patch implantation. Rats were fixed after anesthesia; ejection fraction (EF), fractional shortening (FS), left ventricular end-diastolic volume (LVEDV) and left ventricular end-systolic volume (LVESV) was measured by standard transthoracic echocardiography (EPIQ 5, Philips, Holland) as recommended by the American Echocardiography Association. Ratio of the interventricular septum thickening (ΔIVS), ratio of left ventricular posterior wall thickness (ΔLVPW) and left ventricular mass (LVM) were calculated. Cardiac ultrasonography was performed by the same researcher to avoid measurement errors. All measurements were repeated 3 times.

### Masson's Trichrome Staining

Masson's trichrome staining was used to measure the degree of myocardial fibrosis at 28 days after patch implantation. Rats in each group were anesthetized as above and hearts were exposed and enucleated, fixed with 4% paraformaldehyde and embedded in paraffin. Slices were obtained in the LV transverse direction and Masson's trichrome staining was performed as previously described (14). ImageJ software was used to evaluate percentage collagen volume fraction (CVF%) and left ventricular wall thickness of the infarcted region.

### Immunohistochemistry

Immunohistochemistry was used to detect the expression of type I collagen (Col I) and CD68, with anti-Col1A1(Abcam, Cambridge, MA) and CD68(Abcam, Cambridge, MA) antibody, respectively, according to the manufacturer's instructions. Image J software was used to evaluated the level of Col I deposition and CD68 in the infarcted region.

### Myocardial Stiffness

Myocardial stiffness was measured with a BOSE Electro Force mechanical tester (type:3200). Myocardial tissue was dissected from the infracted zone and prepared for biomechanical tests. The tensile test involved passively stretching the muscle at a constant strain rate to 1.3 times its initial length, at a rate of 0.01 mm/s. Muscle length, width and thickness were measured by using a micrometer and recorded as l, h and d, respectively. After stretching, a stress-displacement curve was obtained based on the raw data recorded by the mechanical tester, and the slope of this line, k, was calculated. Then myocardial stiffness was calculated as E = kl/hd, where E is myocardial stiffness.

### Isolation, Culture, and Identification of Cardiofibroblasts

CFs were isolated from 2- to 3-day-old neonatal rats by using enzyme digestion method. The experimental protocol was approved by the Animals Committee of Xi'an Jiaotong University. In brief, neonatal rat ventricle tissues were separated and fully digested by collagenase II, then purified with differential adhesion method. After 45 min, the supernatant was removed and softly washed with PBS solution twice, then equivalent fresh DMEM/F-12 culture medium containing 10% fetal bovine serum was added and cultured in an incubator with 5% CO_2_ and 95% O_2_ at 37°C. Cultured CFs were identified by immunofluorescence staining of vimentin as described (24).

### Cell Proliferation Assay

Cell counting kit-8 (Abcam, Cambridge, MA) assay was used to observe the cell viability of CFs after treatment with rGO solution. Cultured CFs were seeded in 96-well plates, then exposed to different concentrations of rGO (10, 20, 40, 60, 80, and 100 μg/ml) at 37°C with 5% CO_2_ for 6, 12, 24, and 48 h respectively. Then equivalent CCK-8 was added to each well and 96-well plates were incubated at 37°C for 2 h. The absorbance of each well was recorded at 450 nm by using a Thermo Fisher microplate reader.

### ELISA Assay for Col I and Co1 III

CFs were seeded in 6-well plates with ~10^5^ cells in each well and randomly divided into 4 groups after 2 days of culture, to which were added different intervention factors: control group, angiotensin II, angiotensin II+ rGO (50 μg/ml), angiotensin II+ rGO (100 μg/ml). The concentration of Col I and Col III in the supernatant was measured by using an ELISA assay kit (Shanghai Enzyme-linked Biotechnology Co, Shanghai, China) (*n* = 3 per group) according to the manufacturer's instructions.

### RNA Extraction and Quality Control

Total RNA was extracted from samples of each group by using Trizol reagent (Invitrogen) and the miRNeasy mini kit (Qiagen). RNA quality and quantity were measured by using the NanoDrop spectrophotometer (ND-1000, Nanodrop Technologies, Wilmington, DE, USA) and RNA integrity was determined by gel electrophoresis.

### RNA Sequencing (RNA-Seq)

Total RNA was extracted as above mentioned, 1~2 μg RNA was used to prepare the sequencing library. Total RNA was enriched by oligo magnetic beads; RNA-seq library preparation involved the KAPA Stranded RNA-Seq Library Prep Kit (Illumina), which incorporates dUTP into the second cDNA strand and renders the RNA-Seq library strand-specific. The completed libraries were qualified by using Agilent 2100 Bioanalyzer and quantified by the absolute quantification qPCR method. Before sequencing the libraries, the barcoded libraries were mixed and single-stranded DNA was denatured in 0.1 mM NaOH solution, captured on Illumina flow cell, amplified in situ, and sequenced for 150 cycles for both ends on an Illumina HiSeq instrument. The differentially expressed genes and transcripts were fltered using R package Ballgown. Hierarchical clustering, Gene Ontology and Pathway analysis was performed with the differentially expressed genes in R, Python or shell environment for statistical computing and graphics.

### Real Time-PCR

Total RNA was extracted as above, the reverse transcription reaction involved a 50-ng system of total RNA with a high-capacity cDNA archive kit following the instructions. qRT-PCR involved using the SYBR Green qPCR Kit (TaKaRa,Japan) on a Step-One plus PCR system (ABI, USA). The gene-specific primers and annealing temperatures are in Supplementary Table 1. The results were normalized to GAPDH level, and relative gene expression was measured with the 2^−ΔΔ*Ct*^ method (25).

### Western Blot Analysis

CFs were seeded in 6-well plates and incubated for 24 h at 37°C. After the addition of angiotensin II (10 mM) for 2 h, CFs were treated with different concentrations of rGO solution (50 and 100 μg/ml) for 24 h. The treated CFs were lysed with RIPA buffer, and protein concentrations of cell lysates were quantified with a BCA protein assay kit. Equal amounts of protein (50 μg/lane) were separated on SDS-PAGE (different concentration of gel selected depending on the molecular weight of the target protein) and electro-blotted onto PVDF membranes. After blockade with 8% non-fat milk for 2 h, PVDF membranes were incubated overnight at 4°C with primary antibodies against Yes-associated protein (YAP) (CST, Danvers, MA) (1:1,000), Transcriptional coactivator with PDZ-binding motif (TAZ) (CST, Danvers, MA) (1:1,000), transforming growth factor β1 (TGF-β1) (Abcam, Cambridge, MA) (1:1,000), Samd3(Abcam, Cambridge, MA) (1:1,000), Smad4 (Abcam, Cambridge, MA) (1:5,000), and Col1A1(Abcam, Cambridge, MA) (1:1,000). Then, blots were washed three times with TBST and incubated with the corresponding horseradish peroxidase-conjugated secondary antibody at room temperature for 1 h. Optical density of the bands was scanned by using Tanon-5500 Chemiluminescent Imaging System (Tanon, China). GAPDH was an endogenous control, and data were normalized to GAPDH levels.

### Statistical Analysis

All results are expressed as mean ± standard deviation (SD). For multiple comparisons, one-way ANOVA was used with Bonferroni *post hoc* test. All statistical analyses were performed with SPSS 18.0 for Windows (PASW Statistics, SPSS Inc, Chicago, IL). *P* < 0.05 indicated a statistically significant difference.

## Results

### rGO/Silk Patch Improves Heart Function

**Figure 1:**
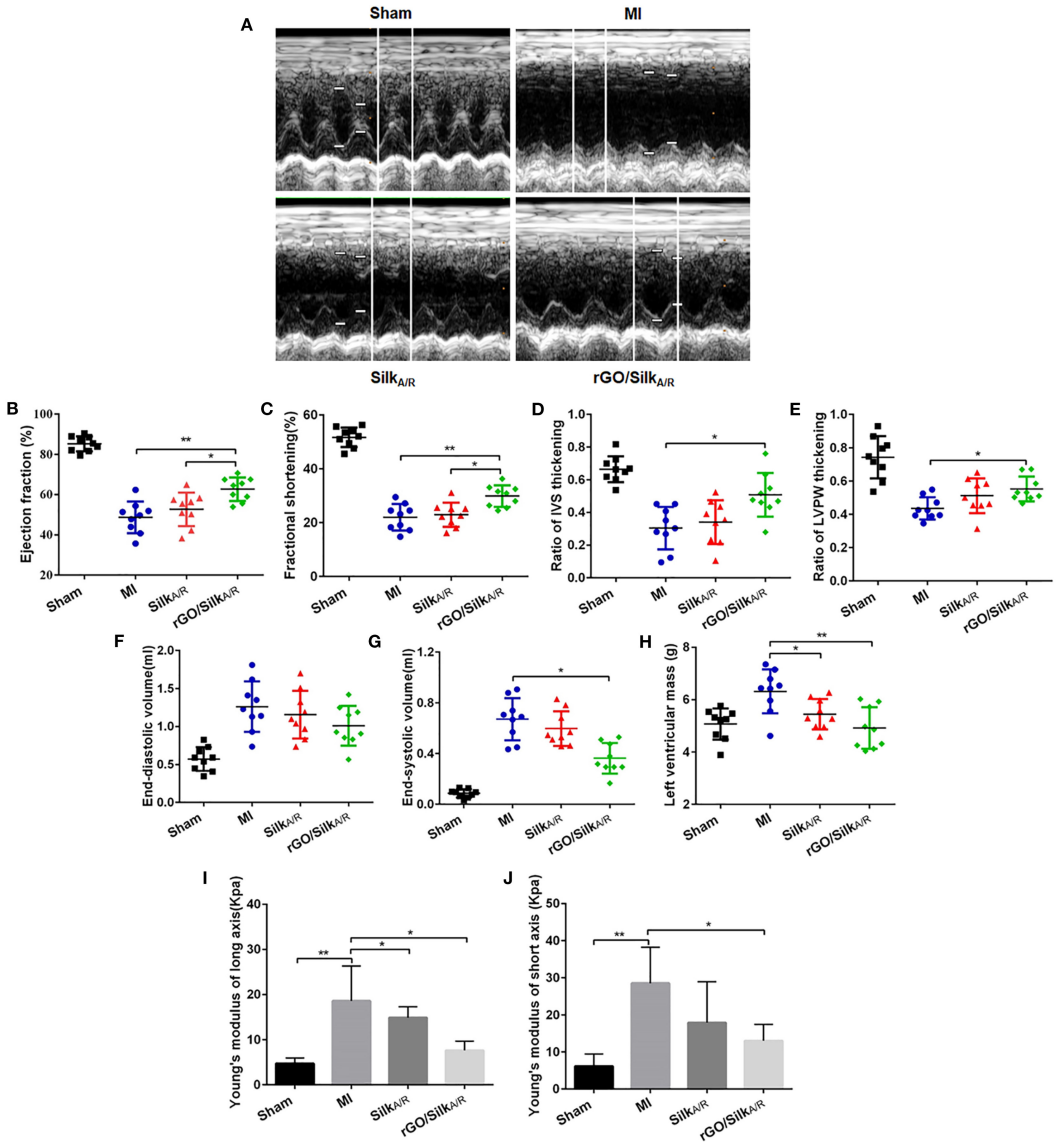
Echocardiographic measurements and biomechanical tests of the effects of rGO/silk cardiac patches on the myocardial functional recovery of infarcted hearts of rats. **(A)** Echocardiography was performed at 4 weeks' post-implantation of patches. Quantification of parameters reflecting blood pumping function, including ejection fraction **(B)**, fractional shortening **(C)**, ratio of interventricular septum (IVS) thickening **(D)** and ratio of left ventricular posterior wall (LVPW) thickening **(E)** at 4 weeks after MI. Quantification of parameters reflecting ventricular remodeling, including left ventricular end-diastolic volume **(F)**, end-systolic volume **(G)** and left ventricular mass **(H)**. Young's modulus both in long axis **(I)** and short axis **(J)** were gauged by an BOSE Electro Force mechanical tester. Data are mean ± SEM from three independent experiments. ^*^P < 0.05, ^**^P < 0.01.

Cardiac systolic function was significantly decreased in infarcted rats as compared with the sham group (Figures 1A–C). After treatment with silk-alone patches, EF and FS were not improved as compared with the MI group, whereas hearts treated with rGO/silk patches showed a significantly higher recovery of cardiac function than MI-alone rats (EF: 62.53±6.23% vs. 48.11 ± 9.78%, *P* = 0.002; FS: 29.98 ± 4.23% vs. 21.38 ± 5.38%, *P* = 0.003). In addition, both ΔIVS and ΔLVPW were increased in the rGO/silk rats as compared with MI-alone rats (Figures 1D,E), we deduced that rGO/silk patches improved the systolic function of infarcted hearts. Furthermore, rGO/silk rats showed improved ventricular remodeling (Figures 1F–H): as compared with MI-alone rats, rGO/silk but not silk-alone rats showed decreased EDV (0.94 ± 0.22 vs. 1.27 ± 0.41 ml, *P* = 0.071) and ESV (0.37 ± 0.13 vs. 0.68 ± 0.27 ml, *P* = 0.014). LVM showed similar trends. Therefore, rGO/silk patches had a better effect on improving cardiac systolic function and ventricular remodeling of infarcted rat hearts than did silk-alone patches.

The stiffness of myocardial tissue of the infarcted zone is measured by passively stretching the muscle at a constant strain rate, which offers a direct approach to understanding intrinsic muscle compliance in hypertrophy and failure (26, 27). Young's modulus is a physical quantity describing the resistance of the solid material to deformation, and its magnitude is inversely proportional to elasticity. MI rats had higher Young's modulus both in the long axis and short axis as compared with sham rats (18.61 ± 6.61 vs. 4.74 ± 1.23 kPa, *P* = 0.005; short axis: 28.53 ± 9.71 vs. 6.13 ± 3.31 kPa, *P* = 0.02) (Figures 1I,J). In the rGO/silk group, the Young's modulus was significantly decreased in both the long axis and short axis as compared with the MI group (long axis: 7.63 ± 2.05 vs. 18.61 ± 6.61 kPa, *P* = 0.02; short axis: 13.03 ± 4.39 vs. 28.53 ± 9.71 kPa, *P* = 0.016). The silk-alone group showed similar results but not statistically significant in the short axis (*P* > 0.05). Therefore, both rGO/silk and silk-alone patches could decrease the Young's modulus of myocardial tissue in the infarcted zone. We speculated that the rGO/silk patch is more effective than the silk-alone patch perhaps because of the action of rGO. This rGO/silk patch provides direct mechanical support to the infarcted area, improves diastolic compliance and reduces wall stress, preventing chamber dilation.

### rGO/Silk Patch Ameliorates Cardiac Fibrosis

**Figure 2:**
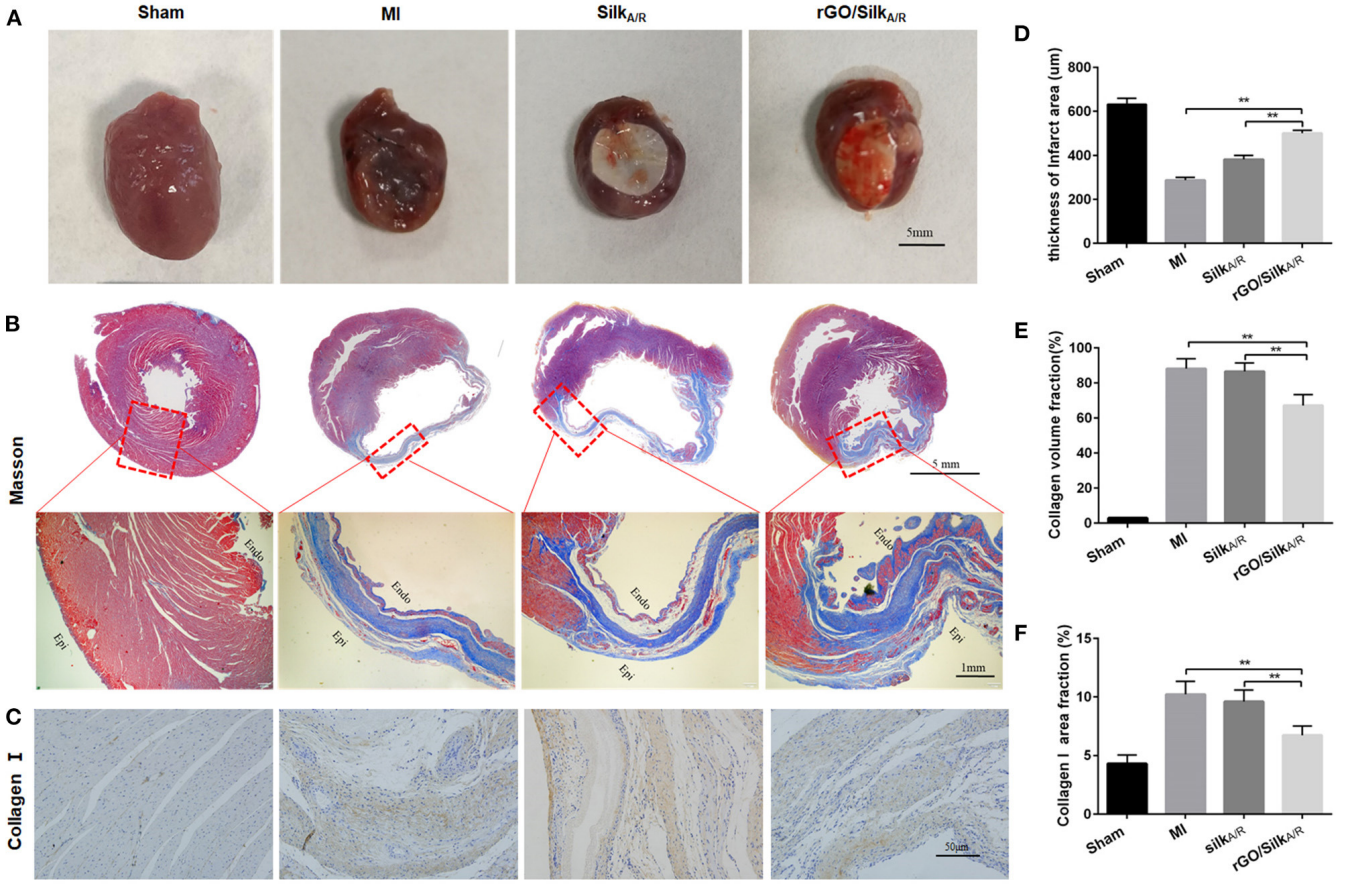
Histological characterization of infarcted hearts. **(A)** Photographs of rat hearts harvested 28 days after implantation. **(B)** Masson's trichrome staining of the heart transections of rats. **(C–F)** Immunohistochemical staining and quantitative analysis of Col1a1 to detect cardiac fibrosis of myocardial tissues. Left ventricle wall thickness **(D)** and collagen volume fraction (CVF%) **(E)** of infarcted area. Data are mean ± SEM from three independent experiments. ^**^P < 0.01.

After echocardiography evaluation, the hearts of each group were harvested (Figure 2A) and Masson's trichrome was used to stain paraffin sections, labeled collagen scar tissue (blue) and cardiac muscle (red). The hearts of the MI group showed the blue color of collagen scar tissue in the infarcted area; the group with rGO/silk patches showed decreased collagen scar and more cardiac muscle (Figure 2B). Then we assessed the LV wall thickness of the infarcted region in each group. rGO/silk patches increased LV wall thickness as compared with the MI and silk-alone group (500.64 ± 13.26 vs. 286.53 ± 13.14 μm, *P* < 0.0001; 500.64 ± 13.26 vs. 381.33 ± 18.3 μm, *P* < 0.0001), with no significant difference between the silk-alone and MI groups (Figure 2D). We further evaluated the collagen volume fraction (CVF%) of the infarcted region (Figure 2E). The rGO/silk patch group had the lowest CVF%, which indicates lower fibrosis of the infarcted zone than the MI group (67.1 ± 6.2 vs. 88.1 ± 5.6, *P* = 0.012), the CVF% for the silk-alone and MI groups was similar (86.5 ± 4.8 vs. 88.1 ± 5.6, *P* = 0.0610.05). These tissue-level histological results suggest that the rGO/silk patches can significantly alleviate the level of cardiac fibrosis, but the mechanism of the rGO/silk patch for ameliorates cardiac fibrosis was unknown.

Type I collagen is the main component of the extracellular matrix of the heart. As compared with the sham group, the MI group showed increased deposition of Col I in the cardiac interstitial space (Figures 2C,F). With rGO/silk patches, deposition of Col I was decreased. Col I area fraction was significantly reduced in the rGO/silk group as compared with MI group (6.73 ± 0.79 vs. 10.22 ± 1.12, *P* < 0.0001), with no difference between the silk patch alone and MI groups, which suggests the potential role of rGO/silk cardiac patches in diminishing the deposition of Col I in the infarcted area. Moreover, all these improvements were more obvious in the rGO/silk than silk-alone group. In addition, both the rGO/silk and silk-alone group showed very good histocompatibility, as measured by immunohistochemical staining with CD68, the specific marker of macrophages to observe the level of chronic inflammation (Supplementary Figure 1).

### rGO Altered CF Cell Viability and Dysregulated Gene Expression

**Figure 3:**
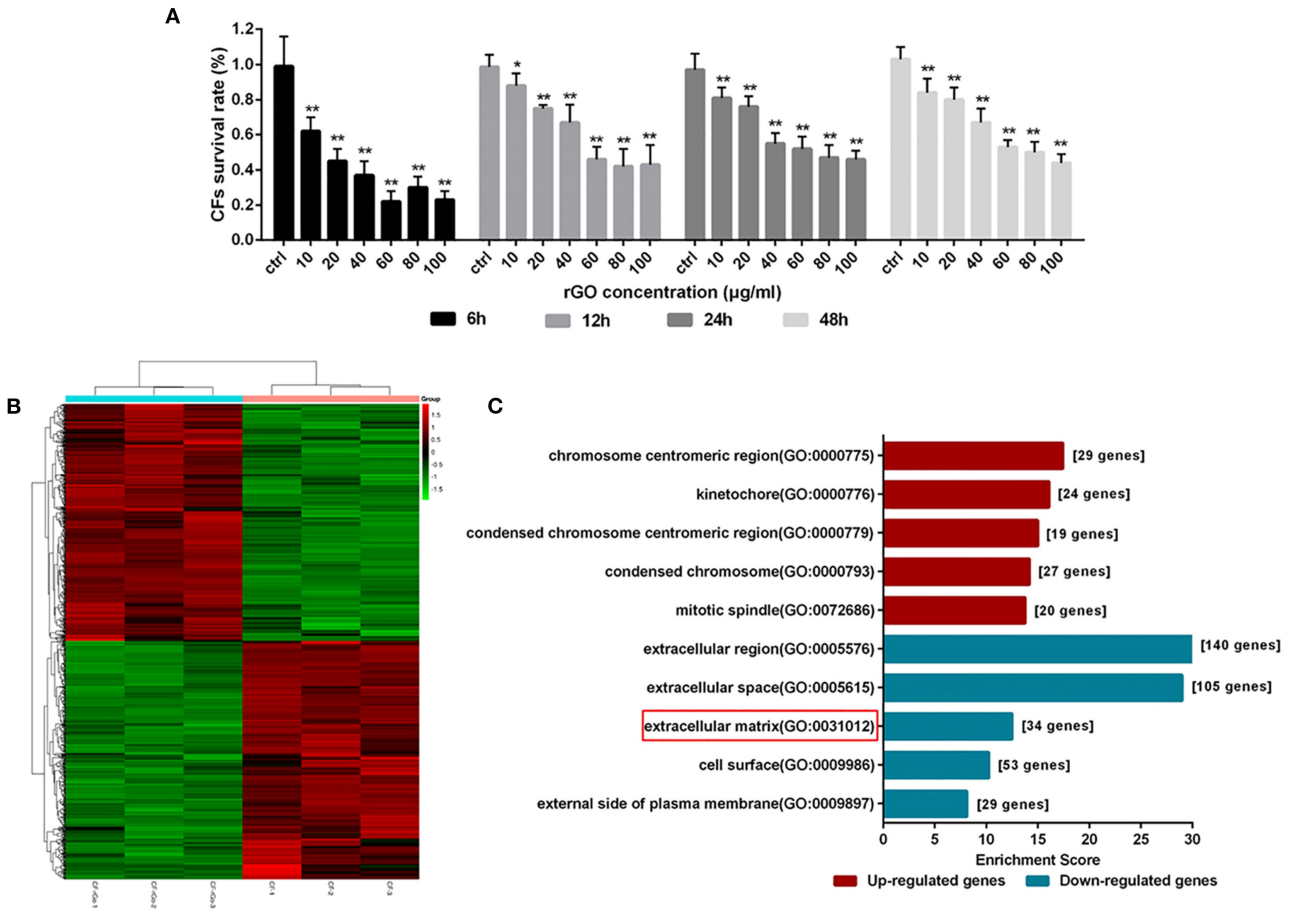
rGO altered cardiofibroblasts (CFs) cell viability and dysregulated gene expression. **(A)** CFs were treated with rGO at different concentrations; cell viability was determined at different times by using CCK-8 assay. **(B)** Heatmap of dysregulated genes. **(C)** GO analysis of differentially expressed mRNAs at the cellular component level. Data are mean ± SEM from three independent experiments. ^*^P < 0.05, ^**^P < 0.01.

To observe the direct effect and potential anti-fibrosis mechanism of rGO on CFs, we used CF viability assay and RNA-seq. CF viability was measured after treatment with rGO at different concentrations (10, 20, 40, 60, 80, and 100 μg/ml). rGO inhibited CF proliferation dose-dependently (Figure 3A). Cell viability was sharply decreased with 40 and 60 μg/ml rGO, and more than 50% of CFs died. Therefore, 50 μg/ml was used as the 50% inhibitory concentration of rGO in the following experiments to investigate its effect on the function of CFs.

To explore the differential expression of genes involved in CFs after rGO treatment, we used RNA-seq to map and analyze the regulatory elements of the genome. A total of 13214 mRNAs were differentially expressed (Figure 3B). At fold change ≥1.5 and *P* < 0.05, 903 differentially expressed genes (DEGs) were detected, including 450 upregulated and 453 downregulated. The functions of the DEGs were predicted by using Gene Ontology (GO) and Kyoto Encyclopedia of Genes and Genomes (KEGG) pathway analyses. At the cellular component level, for the 453 downregulated DEGs, GO terms were mainly related to extracellular region, ECM and cell surface (Figure 3C). Hence, the function change of CFs after intervention of rGO focused on the extracellular region including ECM. Moreover, KEGG pathway analysis showed that those DEGs were involved in signal transduction pathways (Supplementary Figure 2), including IL-17 signaling pathway (PATH: rno04657), cytokine receptor interaction (PATH: rno04060) and hypertrophic cardiomyopathy (PATH: rno05410).

### The Mechanism of the rGO/Silk Patch in Ventricular Fibrosis After MI

**Figure 4:**
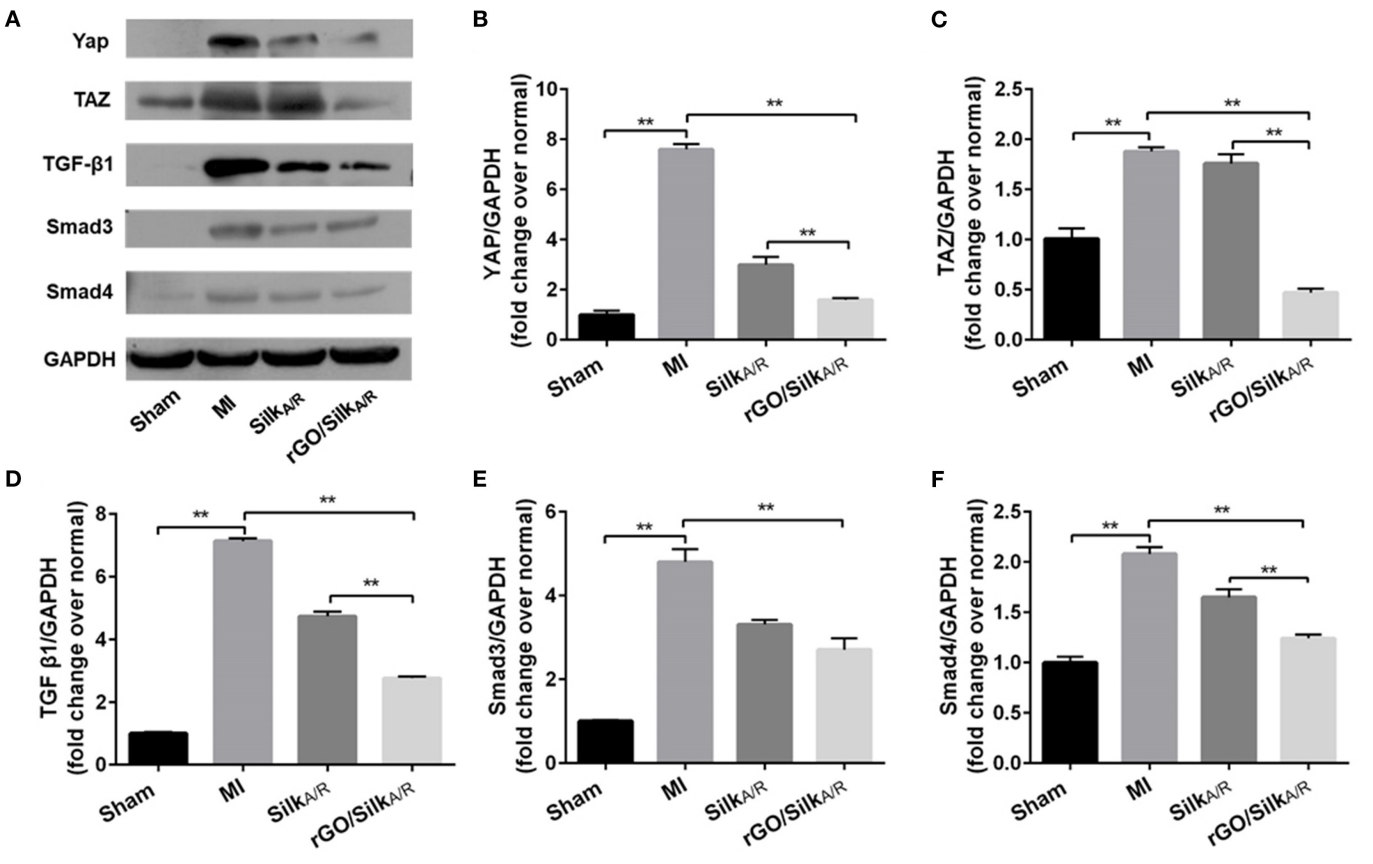
Western blot analysis of the expression of YAP/TAZ and canonical TGF-β1/Smads signaling pathway in infarcted region. Myocardial fibrosis (YAP, TAZ, TGF-b1, Smad3 and Smad4) in rat myocardium was examined at 28 days' post-surgery. Data are mean ± SEM from three independent experiments. Data are mean ± SEM from three independent experiments. ^**^P < 0.01.

To validate the potential mechanism of the rGO/silk patch in reducing the cardiac fibrosis of the infarcted zone, we observed the protein expression of the canonical TGF-β1/Smads signaling pathway and mechanical stress-related signaling pathway, the Hippo pathway (specifically including YAP/TAZ), by western blot analysis. The expression of the TGF-β1/Smads signaling pathway and YAP/TAZ genes in the silk-alone group was downregulated as compared with the MI group (Figures 4A–C), which suggests that the silk patch may activate Hippo signaling via mechanical support, then downregulate the expression of the TGF-β1/Smads signaling pathway. Furthermore, the rGO/silk patch significantly reduced the expression of YAP/TAZ and myocardial fibrosis biomarkers TGF-β1, Smad3, and Smad4 in the infarcted rat myocardium as compared with silk alone (Figures 4A–F), so the rGO/silk patch had a better effect on reducing the expression of the TGF-β1/Smads signaling pathway than silk alone. rGO may have direct effect on the TGF-β1/Smads and YAP/TAZ pathways.

**Figure 5:**
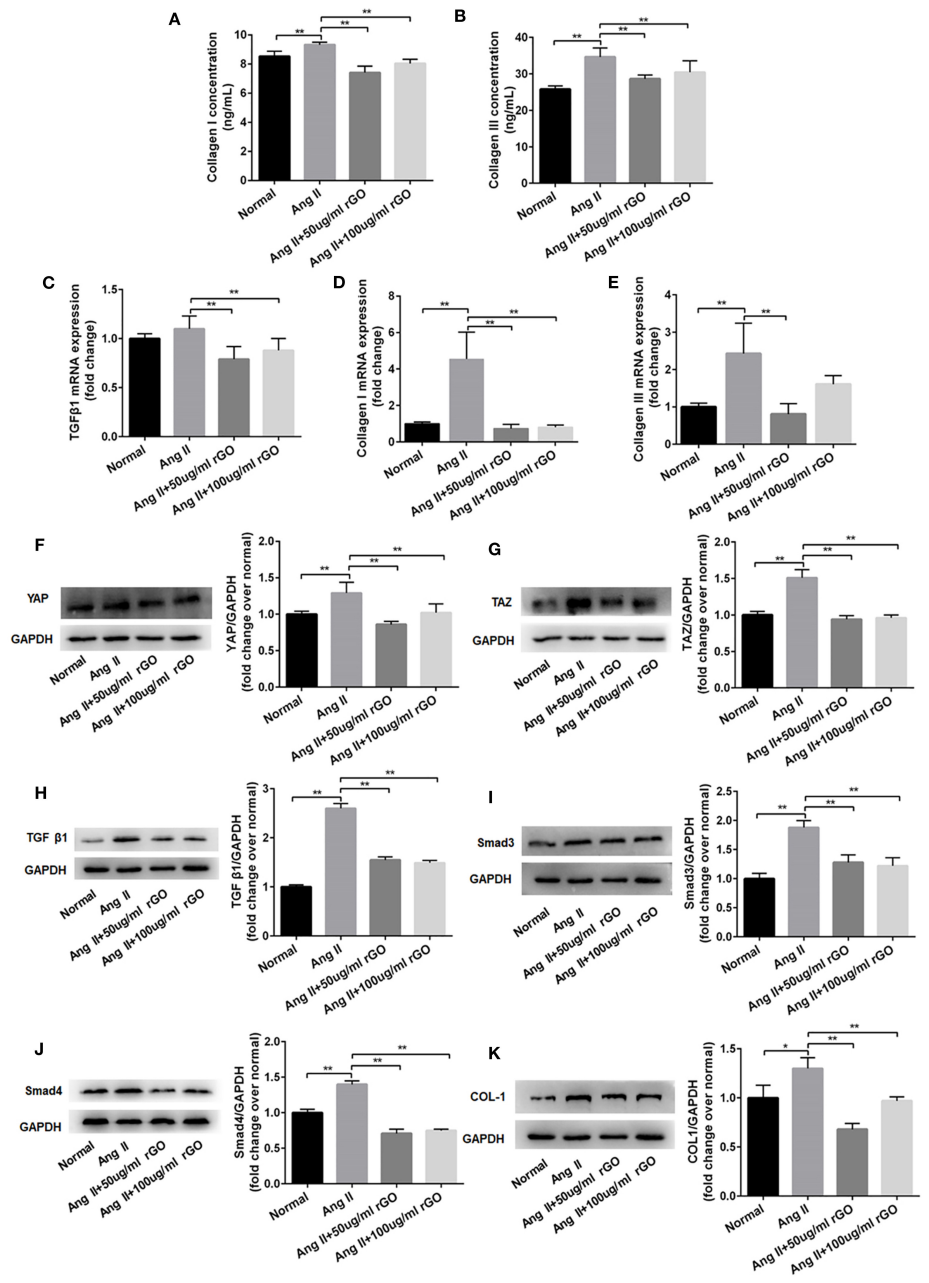
rGO decreased the secretion of Col I and Co1 III in cardiofibroblasts (CFs) through Yap/Taz-TGFβ1/Smads signaling. **(A,B)** Effect of rGO on the secretion of Col I and Co1 III by CFs after angiotensin II (Ang II) treatment; CFs were treated with Ang II and rGO at 50 or 100 μg/ml for 24 h. **(C–E)** Effect of rGO on the mRNA expression of Col I, Co1 III and TGF-β1. **(F–K)** Western blot analysis of the signaling pathway (YAP, TAZ, TGF-b1, Smad3, Smad4 and Col I) in CFs stimulated with Ang II and with rGO. Data are mean ± SEM from three independent experiments. *P < 0.05, **P < 0.01.

To further test this hypothesis, we observed the angiotensin-induced expression of Col I and Co1 III by ELISA and real-time PCR after rGO intervention. As compared with the angiotensin II control group, after treatment with different concentrations of rGO (50 and 100 μg/ml), the secretion was decreased but not dose-dependently for Col I (9.32 ± 0.18 vs. 7.41 ± 0.45 ng/ml, *P* < 0.001; 9.32 ± 0.18 vs. 8.04 ± 0.29 ng/ml, *P* < 0.001) and Col III (34.65 ± 2.46 vs. 28.68 ± 1.01 ng/ml, *P* < 0.001; 34.65 ± 2.46 vs. 30.47 ± 3.10 ng/ml, *P* = 0.006) (Figures 5A,B). Angiotensin II upregulated the mRNA level of Col I, Col III and TGF-β1 (*P* < 0.01) (Figures 5C–E). After treatment with rGO (50 and 100 μg/ml), the mRNA levels showed similar decreased levels as with ELISA: Col I (4.55 ± 1.47 vs. 0.74 ± 0.23, *P* < 0.0001; 4.55 ± 1.47 vs. 0.81 ± 0.12, *P* < 0.0001), Col III (2.43 ± 0.81 vs. 0.81 ± 0.28, *P* = 0.001; 2.43 ± 0.81 vs. 1.61 ± 0.23, *P* = 0.063). Moreover, the mRNA level of TGF-β1 was decreased (1.1 ± 0.1 vs. 0.79 ± 0.13, *P* = 0.001; 1.1 ± 0.1 vs. 0.88 ± 0.12, *P* = 0.009) as compared with the angiotensin II control.

**Figure 6:**
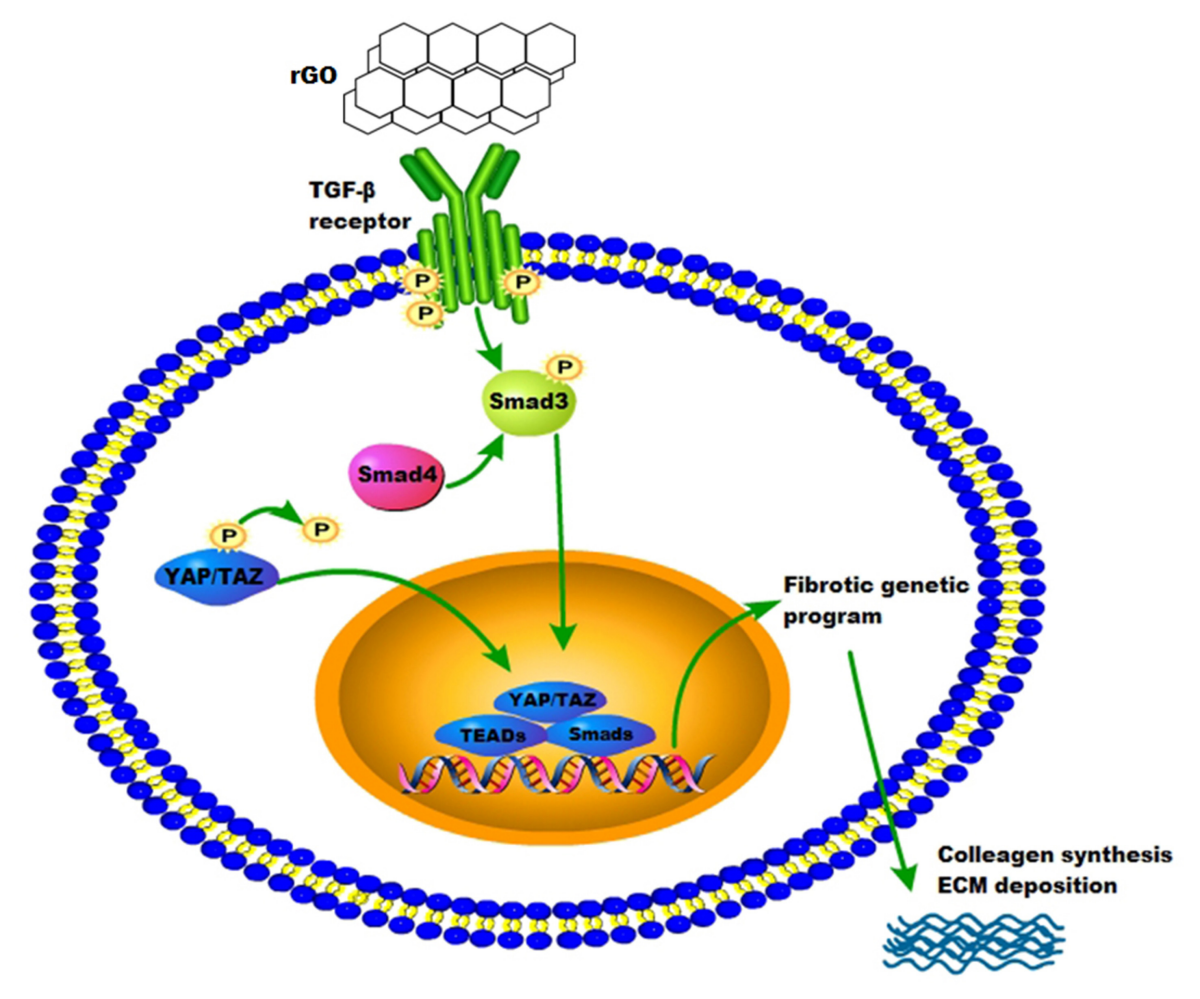
Proposed model of the possible mechanisms of rGO in reducing myocardial fibrosis.

To confirm the molecular mechanism of rGO reducing cardiac fibrosis, we compared the protein levels of YAP/TAZ and canonical TGF-β1/Smads signaling pathway at the cytological level. The protein levels of YAP/TAZ, TGF-β1, Smad3, Smad4 and Col I was increased after angiotensin II treatment (*P* < 0.05) (Figures 5F–K). With rGO treatment (50 and 100 μg/ml), the protein levels of the above genes were significantly decreased but not dose-dependently, and the differences were statically significant (*P* < 0.05). Thus, rGO reduced the cardiac fibrosis possibly through YAP/TAZ and the TGF-β1/Smads signaling pathway. Our data suggested that the addition of rGO regulated the expression of collagen. The introduction of rGO could promote the nuclear translocation of YAP/TAZ, which subsequently regulated the transcription of the fibrosis-related gene TGF-β. The proteins downstream of TGF-β, Smad proteins, activated the fibrotic genetic program to regulate collagen synthesis and ECM deposition (Figure 6).

## Discussion

In the present study, we constructed a silk-derived nanofiber material incorporating rGO and used this rGO/silk cardiac patch to repair rat hearts after AMI. This reduced GO functionalized silk biomaterial showed important therapeutic effects by improving cardiac function and attenuating cardiac fibrosis of infarcted hearts. We further revealed the roles of rGO/silk in reducing fibrosis and inhibiting the secretion of Col I and Co1 III, which provides new insights into the potential molecular mechanism of the rGO/silk cardiac patch but also helps explain the phenomenon of myocardial functional recovery in infarcted hearts.

Cardiac patches based on synthetic scaffolds are becoming a new strategy for treating AMI (28, 29). Previous studies have investigated implantation of different types of cardiac patches in AMI animal hearts, with positive results including improved cardiac function, neovascularization and attenuated fibrosis (30–32). The biology and mechanics of healing infarcted hearts are complex; mechanical support is the most common explanation for cardiac patches improving cardiac function and ventricle remodeling *in vivo*. Previous studies showed that treating MI rat hearts with appropriate mechanical supports, such as a woven nylon cardiac restraints or poly(l-lactide-co-ε-caprolactone) patches, can also reduce LV diameter and improve some cardiac functions (12). By mechanically reinforcing the infarcted area, cardiac patches decrease wall stress in the infarct and adjacent border zone, which can improve pump function and reduce LV dilation (2). Our rGO/silk patch had not only excellent mechanical but also special biological properties, which could restore cardiac function and reduce fibrosis of the rat heart after AMI.

Silk fibroin is one of the major proteins forming the silkworm cocoon, which has shown excellent biocompatibility, biodegradability and mechanical properties, thus silk fibroin is regard as a promising biomaterial in cardiac tissue engineering (33, 34). SF has been applied in various forms of biopolymer-based composites as cardiac patches and has demonstrated ideal efficacy (14). These scaffolds fabricated by SF can mimic the natural extracellular matrix (ECM) of hearts and provide mechanical support for the recovery after myocardial infarction. However, simple SF scaffolds are limited by poor biological functions Therefore, we incorporate the rGO/silk fibroin-modified nanofibrous patches. The rGO/silk patch is a bi-layered scaffold with isotropic mechanics, incorporating an epicardial-facing cardiac rGO-enriched layer. With the addition of rGO, the beneficial effects were significant in rat hearts with a rGO/silk patch vs. a silk-alone patch. However, as we previously showed, Young's moduli showed no significant change between silk alone and rGO/silk biomaterials (23), so the beneficial effects are not due to different mechanical characteristics. We speculated that rGO may magnify the effect of silk, which reduced cardiac fibrosis by mechanical support.

rGO has attracted great attention in tissue engineering because of its excellent physical and chemical properties including electrical conductivity and mechanical properties and also multiple biological functions to promote the adhesion, growth, proliferation and differentiation of various cells including neural, embryonic, pluripotent and mesenchymal stem cells (35–37). Lee et al. found that rGO/hydroxyapatite matrixes promoted osteogenesis of MC3T3-E1 pre-osteoblasts and induced new bone formation, which has potential application in future bone regenerative medicine (38). A similar repair effect of rGO was found in nerve regeneration and skin tissue engineering (37, 39, 40). In a related study, rGO and its derivatives were used in cardiac tissue engineering, which showed good electric connection between healthy myocardium and cardiomyocytes in the infarcted zone and enhanced cell–cell coupling (41–45). Furthermore, Norahan and co-workers demonstrated that rGO-incorporated collagen scaffolds could improve mechanical properties and cell viability with increasing cardiac gene expression. Also, rGO coating showed antibacterial activity for cardiac patch application (46). In our study, the rGO/silk patch had effects on macrophage, which reflects chronic inflammation by immunohistochemical staining with CD68. Both expression level of CD68 and investigation of differentially expressed mRNAs in cardiac fibroblasts indicated that rGO/silk patch induced infection/immunity and pro-inflammation. A related study also found that the biological application of GO in timely modulation of the immune environment in MI for cardiac repair (47), so the anti-inflammation role of rGO maybe another reason for myocardial functional recovery in infarcted hearts. Additionally, the rGO/silk patch significantly decreased CVF% at 28 days post-MI in a rat model, similar effects as with angiotensin-converting enzyme 1, recognized as a good inhibitor of reverse ventricular remodeling (48). However, the molecular mechanism of rGO reducing fibrosis was still unknown.

After AMI, CFs play a critical role in cardiac remodeling by synthesizing and depositing ECM, communicating with myocytes and other cells. Activation of the renin-angiotensin aldosterone system and release of TGF-β induces conversion of fibroblasts into myofibroblasts, promoting deposition of ECM proteins (4). Related studies revealed that fibroblast differentiation arrest was mediated by the Hippo–YAP pathway, which regulated ECM composition and vascular remodeling during heart development and restore cardiac function (49, 50). Recent studies suggested that rGO and its derivatives could regulate other cell biological functions through TGF-β signaling. Li et al. found that graphene could trigger apoptosis of macrophages by activating the mitochondrial pathway via mitogen-activated protein kinase and TGF-β signaling (51). Also, rGO triggers specific biochemical and biological responses via TGF-β signaling. rGO induced neuronal differentiation by affecting YAP/TAZ localization outside the nuclei and increasing the level of neuronal differentiation marker (52). In this study, the expression of the canonical TGF-β1/Smads signaling pathway and YAP/TAZ in the infarcted rat myocardium was markedly decreased with the rGO/silk patch as compared with the MI and silk patch group. On RNA-seq, the expression of the TGF-β1/Smads signaling pathway in CFs was decreased after rGO treatment. Furthermore, the protein expression of the TGF-β1/Smads signaling pathway and YAP/TAZ in CFs stimulated by angiotensin II and with rGO treatment was reduced. Thus, rGO may play a major role in reducing infarct fibrosis via YAP/TAZ genes and the TGF-β1/Smads signaling pathway, but further study is needed to determine the mechanism of rGO regulating the expression of YAP/TAZ genes.

In this study, we have shown the importance of rGO/silk patch to repair the infarcted myocardium and its potential mechanism of the anti-fibrosis effect, there still exist some limitations that require further investigations. First, the cardiac patch was applied on the epicardium through suturing, which limits clinical translation and applications. second, combining the rGO/silk patch with some stem cells which has shown good therapeutic effects for MI may promote the therapeutic effectiveness. Third, the mechanisms for the observed effects of rGO/silk patch in this study are not fully understood and deserve further investigations.

## Conclusion

rGO/silk, magnifying the effect of the silk-alone patch, improved cardiac function and reduced cardiac fibrosis in heart tissue after AMI. The mechanism of the anti-fibrosis effect may be rGO regulating the Yap/Taz-TGFβ1/Smads signaling pathway in CFs. rGO could be a potential candidate in cardiac tissue engineering and cardiac patch therapeutic strategies.

## Data Availability Statement

The data presented in the study are deposited in the GEO repository, accession number GSE179878.

## Ethics Statement

The animal study was reviewed and approved by the Institutional Animal Care Committee at Xi'an Jiaotong University.

## Author Contributions

DG and XZ led the project and supervised the experiments. YF, GZ, MX, XX, and LY conducted experiments and fulfilled data analysis and performed the bioinformatics work. YF, YM, and MQ discussed the results. YF wrote the manuscript. All authors contributed to the article and approved the submitted version.

## Conflict of Interest

The authors declare that the research was conducted in the absence of any commercial or financial relationships that could be construed as a potential conflict of interest.

## Publisher's Note

All claims expressed in this article are solely those of the authors and do not necessarily represent those of their affiliated organizations, or those of the publisher, the editors and the reviewers. Any product that may be evaluated in this article, or claim that may be made by its manufacturer, is not guaranteed or endorsed by the publisher.

## Abbreviations

AMI: acute myocardial infarction
CFs: cardiofibroblasts
CVF: collagen volume fraction
DEGs: differentially expressed genes
ECM: extracellular matrix
EF: ejection fraction
FS: fractional shortening
GO: gene ontology
LVEDV: left ventricular end-diastolic volume
LVESV: left ventricular end-systolic volume
LVM: left ventricular mass
KEGG: kyoto encyclopedia of genes and genomes
rGO: reduced graphene oxide
RNA-seq: RNA sequencing
SF: Silk fibroin
SD: standard deviation
TAZ: transcriptional coactivator with PDZ-binding motif
TGF-β1: transforming growth factor β1
YAP: yes-associated protein
ΔIVS: ratio of the interventricular septum thickening
ΔLVPW: ratio of left ventricular posterior wall thickness.
