# Supplementary material for: rGO/Silk Fibroin-Modified Nanofibrous Patches Prevent Ventricular Remodeling via Yap/Taz-TGFβ1/Smads Signaling After Myocardial Infarction in Rats
Source: Front Cardiovasc Med. 2021 Aug 16;8:718055. doi: 10.3389/fcvm.2021.718055 (PMC8415403; doi:10.3389/fcvm.2021.718055)
Supplement: Supplementary file 1 [file Data_Sheet_1.docx]

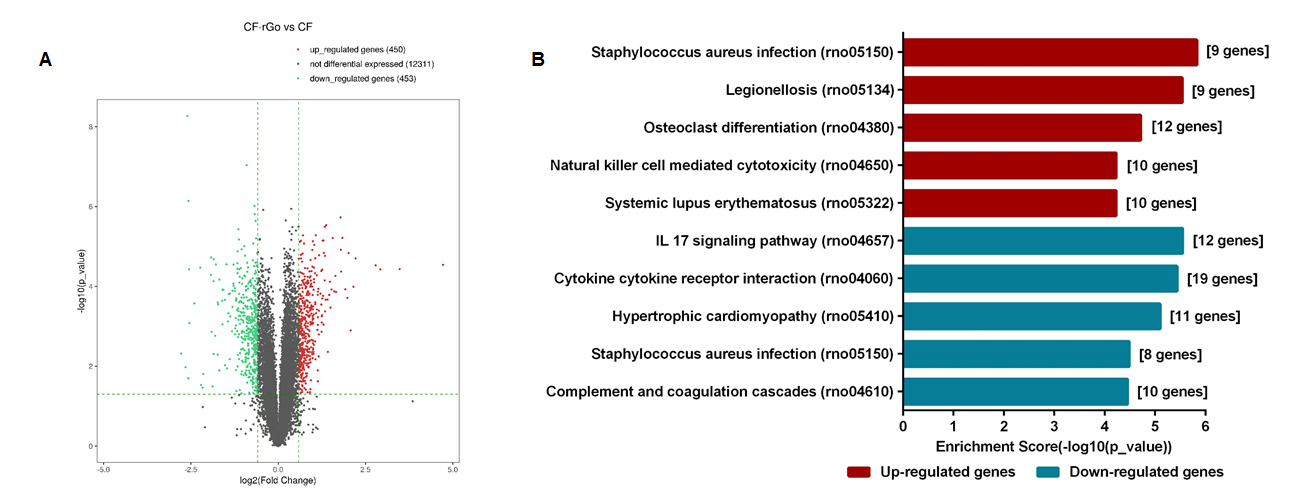
**Figure S1:** Immunohistochemical staining for CD68, a specific marker of macrophages, used to detect chronic inflammation of myocardial tissues, which reflects chronic inflammation at 28 days’ post-surgery. Data are mean ± SEM from three independent experiments. ns, not significant
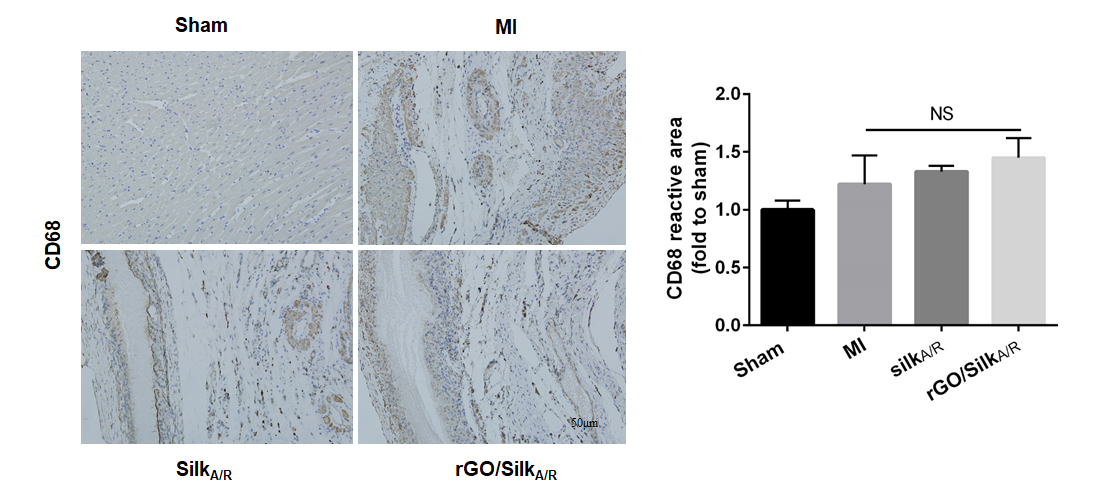
.

**Figure S2:** The functions of differentially expressed mRNAs predicted by KEGG pathway analyses. (A) Volcano plots showing differentially expressed genes in CFs after rGO intervention compared to controls. (B) KEGG pathway analysis of differentially expressed mRNAs.

**Tables**

Table S1. Primers used in this study

| Primer | Type | Primer sequence（5′→3′） |
| --- | --- | --- |
| Col I | F | TGCCGTATGGASCTCAAGATG |
|  | R | CACAAGCGTGCTGTAGGTGAT |
| Col III | F | AGATCATGTCTTCACTCAAGT |
|  | R | TTTACATTGCCATTGGCCTAG |
| TGF-β1 | F | CACCTGCAAGACCATCGACA |
|  | R | CATAGTAGTCCGCTTCGGGC |
| GAPDH | F | AGTTCAACGGCACAGTCAAG |
|  | R | TACTCAGCACCAGCATCACC |
